# Impact of Increasing Antenna Model Complexity on Microwave Tomography Using DBIM

**DOI:** 10.3390/s26113517

**Published:** 2026-06-02

**Authors:** Thomas Vasileiou, Maria Koutsoupidou, Panagiotis Kosmas

**Affiliations:** 1 Meta Materials Europe, 15123 Marousi, Greece; mar.koutsoupidou@gmail.com; 2 Institute of Informatics & Telecommunications, National Center for Scientific Research “Demokritos”, 15310 Agia Paraskevi, Greece

**Keywords:** antenna modeling, calibration, distorted Born iterative method (DBIM), finite-difference time-domain (FDTD), microwave tomography, numerical study

## Abstract

In microwave tomography (MWT), reconstruction accuracy is challenged by modeling error, namely the mismatch between the numerical representation and the actual experiment. Accurate antenna modeling is perceived as an important step toward reducing this error, but the actual benefit of increasing antenna model complexity has not been analyzed in the literature. This work fills this gap by conducting a rigorous numerical analysis of the issue using two popular algorithms for its study: the finite-difference time-domain (FDTD) method for antenna and forward-problem modeling, and the distorted Born iterative method (DBIM) for implementing the iterative inversion algorithm. We consider various FDTD tools of increasing complexity to improve the agreement between the FDTD forward solver and an accurate numerical model implemented in commercial software. After validating these models for different antennas, we perform reconstructions for a stroke-detection scenario. Our results show that in a practical setting, sophisticated antenna modeling in the forward solver does not necessarily improve reconstruction accuracy for monopole-type antennas widely used in MWT. Our model-error analysis confirms that calibration is always necessary in practice and that its impact supersedes efforts to model the antenna more faithfully.

## 1. Introduction

Microwave imaging (MWI) for biomedical applications [1,2] is a non-invasive diagnostic technique that exploits differences in the dielectric properties of biological tissues. The use of low-power, non-ionizing electromagnetic energy, combined with the affordability and compactness of the required devices, makes MWI a promising complementary tool to X-rays and magnetic resonance imaging (MRI) for detecting conditions such as cancer and stroke, as well as for continuously monitoring disease progression.

However, significant improvements in accuracy and sensitivity are necessary for the integration of MWI into clinical practice. First, the development of microwave tomography (MWT) hardware must minimize the mismatch between the experimental and modeled data. Methods in this direction include the careful selection of the coupling medium to enhance signal quality and/or penetration [3,4,5,6], calibration of the setup [1,7,8,9], and characterization of the near-field radiation pattern of the antennas [10]. For image reconstruction, MWT inversion algorithms include various nonlinear and iterative techniques, which can yield comparable results [11]. The incorporation of spatial priors [12], machine learning [13,14,15], and multiple-frequency data [16] can increase the accuracy and stability of MWT reconstructions relative to those achieved using traditional regularization techniques [17].

The mismatch between the experimental data recorded by the imaging setup and the numerical data produced by its model and forward solver is arguably the most important challenge in MWT. This mismatch is attributed to many factors that are difficult to control or model (e.g., thermal noise and radiation from cables feeding the system’s antennas). Beyond thermal noise and cable radiation, the mutual coupling between densely packed antenna elements creates complex electromagnetic interactions that are often oversimplified in forward solvers to maintain computational efficiency, thereby requiring additional image-enhancement algorithmic tools [18]. Attempts to address model errors include the use of machine learning for calibration [19] or specialized calibration matrices to “bridge the gap” between raw *S*-parameters and the idealized field distributions required by inversion algorithms [20]. In contrast, antenna mismatch errors can be addressed using sophisticated but mathematically complex and/or computationally intensive antenna models in the forward solver.

Although accurate antenna modeling has been proposed to reduce model error and improve reconstruction accuracy [21], to the best of our knowledge, there have been no systematic numerical studies on the impact of increasing antenna model complexity. This work aims to fill this gap by comparing the accuracy and computational burden of MWT reconstructions incorporating antenna models with increasing complexity toward more faithful representations of their respective prototypes. To this end, our study considers two commonly used antennas in MWI as elements of our MWT system, a simple dipole and a printed monopole, as well as a patch antenna designed to test a different radiation profile. We develop models of increasing complexity for these antennas and perform reconstructions based on these models in the forward solver. The reconstructions are based on a three-dimensional (3D) implementation of the distorted Born iterative method (DBIM) [16,22], while the forward solver used to simulate the antennas is based on the finite-difference time-domain (FDTD) method. We quantify and compare the results using reconstruction errors and imaging runtimes as metrics of accuracy and computational burden, respectively.

The remainder of this paper is organized as follows. We present the background theory in Section 2.1 and detail antenna modeling and validation in Section 2.2. We describe the numerical testbeds and experiments in Section 2.3, and we present and analyze the results in Section 3. We discuss our findings and conclude this paper in Section 4.

## 2. Materials and Methods

### 2.1. Background

**Figure 1:**
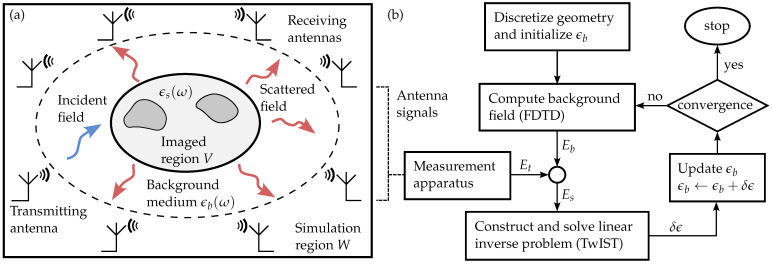
Schematic of the MWT procedure via the distorted Born approximation. (**a**) MWT setup with a fixed array of Tx–Rx pairs. The (inhomogeneous) background has relative permittivity $\epsilon_{b}\left(\omega \right)$, and the imaged region includes a “target region” with permittivity $\epsilon_{s}\left(\omega \right)$ and part of the background region, which is updated at every iteration. (**b**) Flow chart of the DBIM algorithm. The dielectric-constant update δϵ is based on the scattered electric field $E_{s}$, which is the difference between the total $E_{t}$ and the background $E_{b}$ electric fields.

In the inverse scattering problem, we seek to determine the position and value of the complex relative permittivity $\epsilon_{s}$ of the scattering system embedded in a background medium with known complex relative permittivity $\epsilon_{b}$. A set of field measurements is taken at several observation points (Rxs), while a known source (Tx) illuminates the domain of interest *V*, as shown in Figure 1a. The estimate of $\epsilon_{s}$ is constructed from the scattered electric field $E_{s}$; that is, the difference between the total electric field $E_{t}$ measured at the Rx positions and the background (incident) field $E_{b}$, which is calculated from the known distribution of $\epsilon_{b}$.

#### 2.1.1. MWT Problem

In the present study, we employ the DBIM coupled with the two-step iterative shrinkage/thresholding (TwIST) algorithm [16]. The DBIM casts the inverse scattering problem as an iterative optimization problem and recursively finds the optimal solution; at each iteration, an estimate of the dielectric contrast $\delta \epsilon =\epsilon_{b}-\epsilon_{s}$ is obtained as the solution to the linear integral equation

(1) $${\hat{E}}_{s}\left(r_{n},r_{m}\right)={\hat{E}}_{t}\left(r_{n},r_{m}\right)-{\hat{E}}_{b}\left(r_{n},r_{m}\right)=\omega^{2}\mu_{0}\epsilon_{0}\int_{V}{\bar{\bar{G}}}_{b}\left(r_{n},r\right){\hat{E}}_{b}\left(r,r_{m}\right)\delta \epsilon \left(r\right)dr.$$

The above equation relates δϵ to the field $E_{s}$ received by an Rx at position $r_{n}$ when the Tx is located at $r_{m}$. In Equation (1), ${\bar{\bar{G}}}_{b}$ denotes the dyadic Green’s function for the background medium, ω is the frequency of interest, and $\epsilon_{0}$ and $\mu_{0}$ are the permittivity and permeability of vacuum. We use the hat symbol over variables to indicate the frequency-domain representation of a given quantity, e.g., we use ${\hat{E}}_{b}$ for the frequency-domain representation of the field $E_{b}$.

The data-flow diagram of the DBIM algorithm is shown in Figure 1b. We discretize the geometry of interest into equally spaced cells (voxels) and initialize the algorithm with an estimate of $\epsilon_{s}$ or the known value of $\epsilon_{b}$. Each iteration starts with the *forward problem*, which computes the background field ${\hat{E}}_{b}$ for each antenna acting as a Tx. Subsequently, the *inverse problem* involves assembling and solving the discrete version of Equation (1) to determine δϵ. The linear system resulting from the discretization of Equation (1) is severely underdetermined because the number of Tx–Rx pairs is considerably smaller than the number of voxels, *M*, in the domain *V*. Hence, obtaining meaningful values for δϵ requires solving the inverse problem using a linear least-squares (LLS) algorithm that incorporates an explicit or implicit regularization technique. Our implementation iteratively calculates the value of δϵ that minimizes the regularized LLS objective function iteratively using the TwIST algorithm [23]. The $L_{1}$ norm of δϵ, weighted by the constant $\lambda \in R_{>0}$, is added to the LLS loss function to achieve explicit regularization. Moreover, we use an implicit regularization approach through the appropriate termination of the TwIST iterations; specifically, the inner TwIST solver terminates when the relative decrease in the objective function satisfies the tolerance level $e_{r}$, ensuring that the linear sub-problem is sufficiently solved without overfitting to noise [16]. We update the distribution of the known background material with the value $\epsilon_{b}+\delta \epsilon$ after each iteration and repeat the process until either the norm of δϵ or the number of DBIM iterations reaches a predefined threshold.

#### 2.1.2. Forward Solver

**Figure 2:**
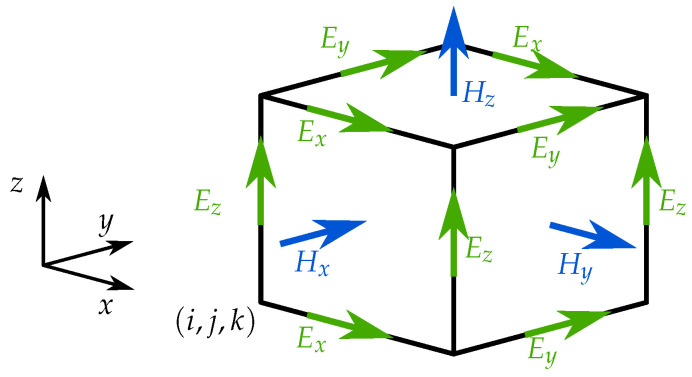
The Yee lattice. Electric ($E_{x}$, $E_{y}$, $E_{z}$) and magnetic ($H_{x}$, $H_{y}$, $H_{z}$) field components in the cell.

We employ the well-known FDTD method [24] to solve the forward problem, which discretizes Maxwell’s equations using finite central differences to approximate the partial derivatives in space and time. The simulation domain is divided into rectangular cells, with the electric and magnetic field components placed on a staggered grid known as the Yee lattice [25]. In particular, the components of the electric field are aligned with the cell edges, and the components of the magnetic field are located at the center of the cell faces, as shown in Figure 2. For imaging applications, the cell is commonly a cube with side length δh, an assumption we adopt in the present study.

The electric and magnetic field components are updated alternately in time; the update of the electric field components is computed from the stored values of the magnetic field, and vice versa. For instance, the electric field in the *z* direction, $E_{z}$, is computed at the time instance $n+\frac{1}{2}$ from the magnetic field components $H_{x}$ and $H_{z}$ as follows:

(2) $$\begin{array}{c} E_{z}{|}_{i,j,k+\frac{1}{2}}^{n+\frac{1}{2}}=C_{a,z}{|}_{i,j,k}E_{z}{|}_{i,j,k+\frac{1}{2}}^{n-\frac{1}{2}}+C_{b,z}{|}_{i,j,k}\left(H_{y}{|}_{i+\frac{1}{2},j,k+\frac{1}{2}}^{n}-H_{y}{|}_{i-\frac{1}{2},j,k+\frac{1}{2}}^{n}\right)\\ \;\;\;\;\;\;\;\;-C_{b,z}{|}_{i,j,k}\left(H_{x}{|}_{i,j+\frac{1}{2},k+\frac{1}{2}}^{n}-H_{x}{|}_{i,j-\frac{1}{2},k+\frac{1}{2}}^{n}\right)-\delta hC_{b,z}{{|}_{i,j,k}J_{s,z}|}_{i,j,k+\frac{1}{2}}^{n-\frac{1}{2}} \end{array}$$

where $C_{a,z}{|}_{i,j,k}$ and $C_{b,z}{|}_{i,j,k}$ are parameters that depend on the background material and the discretization. The subscripts *i*, *j*, and *k* correspond to the cell indices in the *x*, *y*, and *z* directions, respectively. The superscript indicates the associated time instance. The term $J_{s,z}$ is the current density imposed by the source. Likewise, the update for $H_{z}$ reads as

(3) $$\begin{array}{c} H_{z}{|}_{i+\frac{1}{2},j+\frac{1}{2},k}^{n+1}=D_{a,z}{|}_{i,j,k}H_{z}{|}_{i+\frac{1}{2},j+\frac{1}{2},k}^{n}-D_{b,z}{|}_{i,j,k}\left(E_{y}{|}_{i+1,j+\frac{1}{2},k}^{n+\frac{1}{2}}-E_{y}{|}_{i,j+\frac{1}{2},k}^{n+\frac{1}{2}}\right)\\ \;\;\;\;\;\;\;\;\;\;\;\;\;\;\;\;+D_{b,z}{|}_{i,j,k}\left(E_{x}{|}_{i+\frac{1}{2},j+1,k}^{n+\frac{1}{2}}-E_{x}{|}_{i+\frac{1}{2},j,k}^{n+\frac{1}{2}}\right) \end{array}$$

where the parameters $D_{a,z}{|}_{i,j,k}$ and $D_{b,z}{|}_{i,j,k}$ are functions of the permeability of the background material and the discretization. The update equations for the electric and magnetic components in the remaining directions adopt a similar form.

For medical applications, the basic FDTD algorithm is extended to account for material dispersion. Specifically, we consider the single-pole Debye model with loss for all materials; the relative dielectric constant is a function of frequency ω as follows:

(4) $$\epsilon_{b}\left(\omega \right)=\epsilon_{b}^{\prime }-j\epsilon_{b}^{\prime \prime }=\epsilon_{\infty }+\frac{\epsilon_{\Delta }}{1+j\omega \tau_{p}}+\frac{\sigma_{s}}{j\omega \epsilon_{0}}$$

where $\epsilon_{\infty }$ is the relative permittivity at infinite frequency, $\epsilon_{\Delta }$ is the difference between the static relative permittivity and $\epsilon_{\infty }$, $\sigma_{s}$ is the static conductivity, and $\tau_{p}$ is the relaxation constant. The symbol $j=\sqrt{-1}$ denotes the imaginary unit. We denote the real and imaginary parts of $\epsilon_{b}$ by $\epsilon_{b}^{\prime }$ and $\epsilon_{b}^{\prime \prime }$. The effect of dispersion is incorporated into the FDTD equations through the auxiliary differential equation (ADE) method [26,27], which introduces an additional current-density term in Equation (2). In addition, we enforce convolutional perfectly matched layer (CPML) boundary conditions on all external surfaces of the computational domain [28,29]. More details of the FDTD implementation can be found in [30].

#### 2.1.3. Baseline Implementation Assumptions

Solving the 3D forward problem is computationally burdensome and essentially dominates the imaging time for iterative nonlinear algorithms such as the DBIM. We apply several simplifying assumptions in the baseline implementation that either make the imaging problem easier to solve or reduce the imaging time. In later sections, we relax some of these assumptions to facilitate accurate antenna modeling.

First, we choose a *z*-polarized source, which, in combination with negligible cross-polarization scattering effects, reduces all quantities in Equation (1) to scalar form by omitting the *x* and *y* components in the inversion [31]. The Green’s function reduces to a single component, ${\hat{G}}_{b}$, which can be computed from the *z* component of the background electric field ${\hat{E}}_{b}$ as follows:

(5) $${\hat{G}}_{b}\left(r_{n},r\right)=\frac{j}{\omega \mu \delta h}\frac{{\hat{E}}_{b}\left(r\right)}{{\hat{I}}_{s}\left(r_{n}\right)}$$

for a point source with excitation current ${\hat{I}}_{s}$ in a medium with permeability μ. Therefore, we drop the vector notation for the scattered and the background *E* fields for the remainder of this paper for convenience.

With respect to geometry discretization, we assign a single material to each cell. Considering equally spaced cells, this allows one to store a single value per cell for the coefficients $C_{a}{|}_{i,j,k}=C_{a,x}{|}_{i,j,k}=C_{a,y}{{|}_{i,j,k}=C_{a,z}|}_{i,j,k}$ and $C_{b}{|}_{i,j,k}=C_{b,x}{|}_{i,j,k}=C_{b,y}{{|}_{i,j,k}=C_{b,z}|}_{i,j,k}$ in Equation (2), which are independent of the field direction. For the single-pole Debye model with loss, the values become

(6) $$\begin{array}{cc} C_{a}{|}_{i,j,k} & =\frac{2\epsilon_{0}\epsilon_{\infty }{|}_{i,j,k}-\sigma_{s}{|}_{i,j,k}\delta t+\beta_{d}{|}_{i,j,k}\delta t}{2\epsilon_{0}\epsilon_{\infty }{|}_{i,j,k}+\sigma_{s}{|}_{i,j,k}\delta t+\beta_{d}{|}_{i,j,k}\delta t} \end{array}$$

(7) $$\begin{array}{cc} C_{b}{|}_{i,j,k} & =\frac{2\delta t}{\delta h(2\epsilon_{0}\epsilon_{\infty }{|}_{i,j,k}+\sigma_{s}{|}_{i,j,k}\delta t+\beta_{d}{|}_{i,j,k}\delta t)} \end{array}$$

where δt is the simulation time step and $\beta_{d}{|}_{i,j,k}=\left(2\epsilon_{0}\epsilon_{\Delta }{|}_{i,j,k}\right){\left(2\tau_{p}+\delta t\right)}^{-1}$. We point out that considering one material per cell and per direction also reduces the unknown variables in the inverse problem by a factor of three, i.e., to one instead of three per cell. Moreover, we examine non-magnetic materials; therefore, $D_{a}=1$ uniformly and $D_{b}$ adopts the scalar value

(8) $$D_{b}=\frac{\delta t}{\mu_{0}\delta h}.$$

All of these assumptions both simplify the problem and enable various software optimizations that significantly reduce imaging time [30].

FDTD forward solvers can be further simplified by modeling the transmitting antennas as a “soft” point source [32], which occupies a single FDTD cell and imposes a current density $J_{s,z}=I_{s}\delta h^{-2}$ in the *z* direction. In Section 2.2, we present methods that allow detailed antenna representations, which we evaluate against the point-source approximation with respect to image reconstruction quality and computational cost.

#### 2.1.4. Total Field Measurement and Calibration

In practical applications of MWT, antenna signals are recorded by a vector network analyzer (VNA), which provides the scattering parameters (*S*-parameters) for the antennas. The application of DBIM requires the value of the total field in Equation (1); therefore, the captured VNA signals should be converted to a convenient form. To this end, we use the calibrated *S*-parameter, $S_{pq,c}$, from a known reference geometry to scale the measurement [7,8,9], as

(9) $${\hat{E}}_{t}\left(\omega \right)=\frac{S_{pq,m}\left(\omega \right)}{S_{pq,c}\left(\omega \right)}{\hat{E}}_{c}\left(\omega \right)$$

where $S_{pq,m}$ is the recorded *S*-parameter for the unknown geometry to be imaged and the subscripts *p* and *q* define the antenna pair in question. The electric field ${\hat{E}}_{c}$ is calculated using the forward solver for the known geometry of a reference scenario used for calibration (e.g., the antenna array and an empty imaging tank). The calibration process is essential for compensating for offsets between the measured and simulated signals due to modeling errors or manufacturing tolerances.

### 2.2. Antennas and Modeling

In FDTD research, various tools have been developed to enable accurate modeling of the antenna geometry and the feeding mechanism. In the following, we review some of these techniques and compare them against the simple point-source model with respect to image reconstruction accuracy. We tested the validity of our implementation using CST Microwave Studio as a second computational solver that simulates the antenna behavior.

#### 2.2.1. Antenna Selection

**Figure 3:**
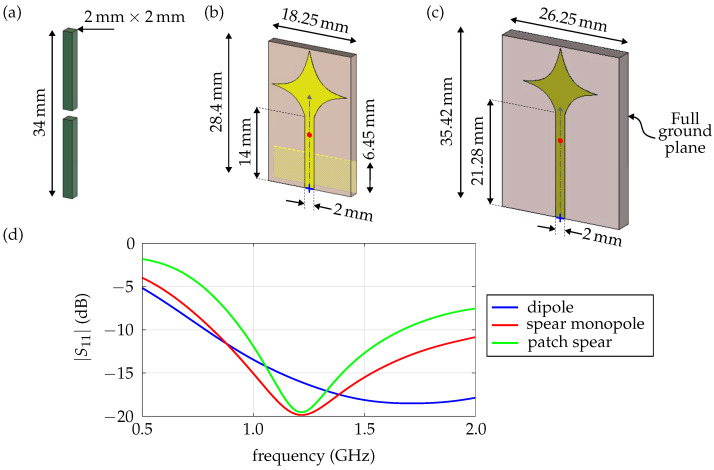
The geometries and dimensions of the three antennas considered in this study: (**a**) dipole antenna, (**b**) spear-monopole antenna, and (**c**) patch-spear antenna. (**d**) Plots of their reflection coefficients, $S_{11}$, when immersed in a 90% glycerol–water mixture. In (**b**,**c**), the blue cross indicates the feed position, and the red dot indicates the location of the equivalent point-source model.

We used three types of antennas in this study: a standard dipole, a planar monopole and its modified version with a ground plane to introduce different radiation properties (see Figure 3). All three antennas operate within the same frequency range and have comparable dimensions. The dipole’s geometry and dimensions allow easy modeling with a coarse cubic mesh. Specifically, we selected a dipole with a rectangular cross-section of 2 mm × 2 mm and a total length of 34 mm, which fits in a space meshed with 2 mm × 2 mm × 2 mm cells (Figure 3a). The planar monopole is a spear-monopole antenna, which has previously been developed for use in a microwave imaging prototype [33] (Figure 3b). The third antenna is a modified version of the spear monopole, redesigned to achieve different radiation characteristics in both directivity and polarization. A full ground plane has been introduced, and the overall dimensions have been scaled to achieve resonance at 1.2 GHz (Figure 3c). Furthermore, the conventional FR4 substrate has been replaced with a thicker alumina substrate of higher dielectric constant to minimize radiation losses associated with substrate modes. This choice is particularly important given the combination of the full ground plane and the high impedance loading of the matching medium.

**Figure 4:**
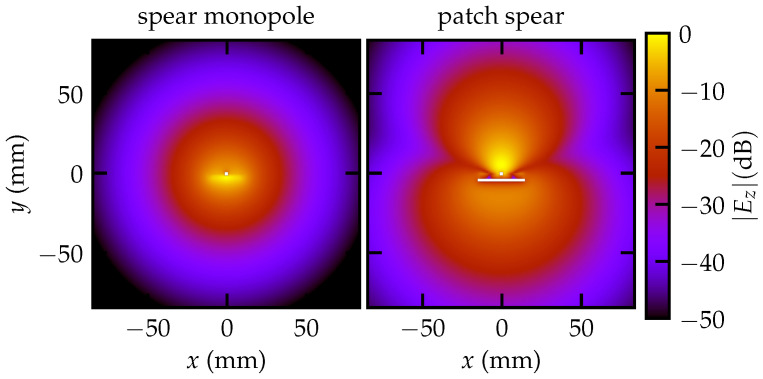
Comparison of the electric field distributions of spear-monopole and patch-spear antennas when immersed in a glycerol–water mixture. The images depict the *z* component of the electric field, $E_{z}$, at 1.2 GHz on a plane perpendicular to the *z* direction, where the *z* axis is parallel to the substrate height. The depicted plane is located at 9 mm above the antenna feed. The values of $E_{z}$ are normalized to the maximum value on the plane.

The reflection coefficient, $S_{11}$, of the three antennas immersed in a dispersive medium with the dielectric properties of a 90% glycerol–water mixture is shown in Figure 3d. This mixture has been proposed as coupling and immersion material in MWI prototypes intended for medical applications [34]. Its dispersive behavior can be modeled by a first-order Debye model with $\epsilon_{\infty }=6.59$, $\epsilon_{\Delta }=29.01$, $\sigma_{s}=0$ S m^−1^, and relaxation time τ=14.3 ns. The electric fields of the spear monopole and the patch spear when immersed in the dispersive medium are depicted in Figure 4, showcasing the distinct radiation characteristics of the two antennas.

#### 2.2.2. Antenna Geometry Modeling

The conventional forward solver adopts a bulk material approach (one material per cell) that does not efficiently support the incorporation of arbitrary antenna geometry. For example, patch antennas include a thin substrate and metal sheets, which are impossible to model under the assumption of a single material assignment for all field directions within each cell. To efficiently model the antennas, we split the FDTD simulation domain into two subregions. The first subregion corresponds to the reconstruction region *V* and retains the assumption of the bulk model. Thus, the inverse problem remains unaffected by the selected antenna model.

The second subregion, *W*, consists of the rest of the domain, where anisotropic material properties are permitted; namely, each cell may contain different Debye model parameters along each direction. Therefore, we store the three FDTD coefficients for each cell in *W*. The use of anisotropic material properties allows for more detailed antenna models. Essentially, we are able to capture surfaces of any material inside the simulation domain, provided the surface faces one of the *x*, *y*, or *z* directions. As an example, for a metal surface on a patch antenna pointing in the *z* direction, we can set $C_{a,x}{|}_{i,j,k}=C_{a,y}{|}_{i,j,k}=1$ and $C_{b,x}{|}_{i,j,k}=C_{b,y}{|}_{i,j,k}=0$ on the respective cells and force the $E_{x}{|}_{i,j,k}$ and $E_{y}{|}_{i,j,k}$ components to be zero, implementing a perfect electric conductor (PEC) surface.

**Figure 5:**
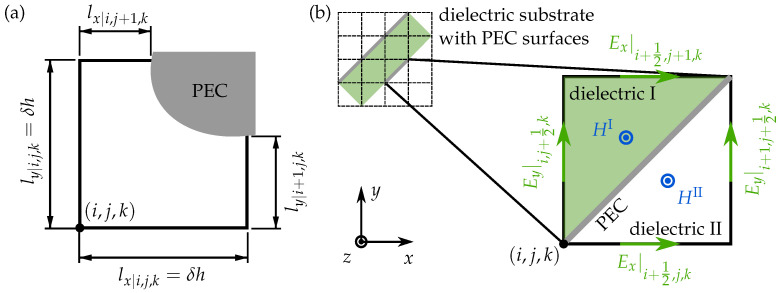
Schematic of conformal cell for modeling curvilinear objects and thin PEC surfaces. (**a**) Cell containing part of the PEC object. (**b**) Example of thin substrate with PEC surface oriented at 45° with respect to the Yee lattice.

In addition, we are able to model curved objects or geometries that do not conform exactly to the staircase discretization of the FDTD grid, by using conformal methods such as the effective subcell-material technique [35,36]. In this conformal method, cells containing interfaces between separate objects are substituted by an effective material, which captures the characteristics of the geometry without refining the mesh. The subcell material technique can model cells that contain a boundary between a PEC structure and a dielectric, as shown in Figure 5a. In the PEC case, the conformal approach requires modifying the *H* field update, as the effective material differs not only with respect to permittivity but also with respect to permeability; for example, the $H_{z}$ field update Equation (3) remains the same, except that

(10) $$D_{b,z}{|}_{i,j,k}=\frac{\delta t}{{\tilde{\mu }}_{z}{|}_{i,j,k}\delta h},\;{\tilde{\mu }}_{z}{|}_{i,j,k}=S_{eff,z}{|}_{i,j,k}\mu_{0}$$

where ${\tilde{\mu }}_{z}{|}_{i,j,k}$ is the effective permeability of the cell in the *z* direction and $S_{eff,z}{|}_{i,j,k}$ is the ratio of the non-PEC surface area to the surface area of the cell face. Moreover, the effective electric field substitutes the original electric field and is obtained by scaling the original field by the inverse edge-length ratio $L_{eff,x}{|}_{i,j,k}=l_{x}{|}_{i,j,k}\delta h^{-1}$, where $l_{x}{|}_{i,j,k}$ is the length of the cell edge outside the PEC region at point (i,j,k) that points in the *x* direction, as shown in Figure 5a. Similarly, the effective Debye model parameters are given by ${\tilde{\epsilon }}_{\infty ,x}{|}_{i,j,k}=\epsilon_{\infty }{|}_{i,j,k}L_{eff,x}^{-1}{|}_{i,j,k}$, ${\tilde{\epsilon }}_{\Delta ,x}{|}_{i,j,k}=\epsilon_{\Delta }{|}_{i,j,k}L_{eff,x}^{-1}{|}_{i,j,k}$, and ${\tilde{\sigma }}_{s,x}{|}_{i,j,k}=\sigma_{s}{|}_{i,j,k}L_{eff,x}^{-1}$, resulting in the modified $C_{b,x}{|}_{i,j,k}$ coefficient in Equation (2):

(11) $$C_{b,x}{|}_{i,j,k}=\frac{2\delta tL_{eff,x}{|}_{i,j,k}}{\delta h(2\epsilon_{0}\epsilon_{\infty }{|}_{i,j,k}+\sigma_{s}{|}_{i,j,k}\delta t+\beta_{d}{|}_{i,j,k}\delta t)}.$$

Similar modifications apply to the remaining field components.

The MWT setup may contain antennas in various orientations that are not aligned with one of the Yee lattice directions. Therefore, the model must accommodate rotated PEC surfaces relative to the main axes to simulate the imaging setup. A conformal method has been proposed to model a PEC layer thinner than the cell size at any orientation relative to the grid cells in [37]. In the present work, we focus on a simplified case, where the PEC structure is an infinitesimally thin plane rotated by ±45° about the *z* axis, as shown in Figure 5b. The PEC surface divides the cell into two triangular prisms, each containing a different dielectric material. To capture the electromagnetic behavior, we divide the magnetic field in the *z* direction into two values, $H_{z}^{I}{|}_{i+\frac{1}{2},j+\frac{1}{2},k}$ and $H_{z}^{\mathrm{II}}{|}_{i+\frac{1}{2},j+\frac{1}{2},k}$, each corresponding to the respective dielectric region. The *H*-field update in Region I is obtained by applying Faraday’s law in the triangular cross-section:

(12) $$H_{z}^{I}{|}_{i+\frac{1}{2},j+\frac{1}{2},k}^{n+1}=\frac{1}{2}D_{a,z}{|}_{i,j,k}H_{z}^{I}{|}_{i+\frac{1}{2},j+\frac{1}{2},k}^{n}+D_{b,z}{|}_{i,j,k}\left(E_{y}{|}_{i,j+\frac{1}{2},k}^{n+\frac{1}{2}}+E_{x}{|}_{i+\frac{1}{2},j+1,k}^{n+\frac{1}{2}}\right)$$

while enforcing a zero tangential electric field on the PEC surface. The *H*-field update for Region II is analogous. For the update of the *E* field components, we use either $H_{z}^{I}$ or $H_{z}^{\mathrm{II}}$, depending on the region (Region I or II) in which the component resides.

We note that the geometry-related calculations involved in the conformal FDTD methods are restricted in *W* and performed during the initialization of the imaging algorithm, thereby excluding them from the iterative part of the imaging procedure. Table 1 summarizes and compares the different antenna modeling approaches used in the present study.

#### 2.2.3. Antenna Feed Modeling

In addition to antenna geometry, accurately capturing the antenna behavior requires modeling of the antenna feed. In typical microwave imaging scenarios, the size of the antenna feed connector is often comparable to δh. Hence, modeling the feed in detail while maintaining a uniform mesh is impractical. Therefore, we approximate the antenna feed with a lumped port; the metallic structure of the antenna is separated at a single FDTD cell with the feed located in the resulting gap [38]. The lumped port spans the gap between the two conductors, for example, the radiating element and the ground plane of a patch antenna. Here, we examine two lumped feed models: the soft source gap feed model and the one-dimensional transmission-line simple-feed model.

The soft source gap feed is analogous to a point source; specifically, the source imposes a current density at the drive point of the antenna. The voltage across a unit cell is related to the electric field as $V_{a}=-E_{z}\delta h$, which is then used to compute the antenna voltage. The antenna current, $I_{a}$, is computed from the discrete form of the line integral

(13) $$I_{a}=\oint_{C}Hdl$$

where the contour C encloses the cross-section of the conductors on each side of the gap.

The transmission-line model connects a virtual transmission line to the drive gap of the antenna [38,39]. An external one-dimensional FDTD lattice, indexed by $k^{\prime }$, emulates a lossless transmission line. The spatial spacing of the lattice is equal to δh. The discretized telegrapher’s equations govern the evolution of the transmission-line voltage *V* and current *I* at point $k^{\prime }$ as

(14) $$\begin{array}{cc} I{|}_{k^{\prime }+\frac{1}{2}}^{n} & =I{|}_{k^{\prime }+\frac{1}{2}}^{n-1}-\frac{C_{u}}{Z_{0}}(V{|}_{k^{\prime }+1}^{n-\frac{1}{2}}-V{|}_{k^{\prime }}^{n-\frac{1}{2}}) \end{array}$$

(15) $$\begin{array}{cc} V{|}_{k^{\prime }}^{n+\frac{1}{2}} & =V{|}_{k^{\prime }}^{n-\frac{1}{2}}-Z_{0}C_{u}(I{|}_{k^{\prime }+\frac{1}{2}}^{n}-I{|}_{k^{\prime }-\frac{1}{2}}^{n}) \end{array}$$

where $Z_{0}$ is the characteristic impedance, $C_{u}=\frac{u_{p}\delta t}{\delta h}$, and $u_{p}$ is the phase velocity within the transmission line.

The transmission-line feed relocates the excitation from the 3D FDTD lattice to the point $k_{exc}^{\prime }$ on the line. We implemented a one-way injector to insert the incident wave toward the 3D FDTD lattice with minimal reflections in the opposite direction. The equations at $k_{exc}^{\prime }$ become

(16) $$\begin{array}{cc} I{|}_{k_{inj}^{\prime }+\frac{1}{2}}^{n} & =I{|}_{k_{inj}^{\prime }+\frac{1}{2}}^{n-1}-\frac{C_{u}}{Z_{0}}(V{|}_{k_{inj}^{\prime }+1}^{n-\frac{1}{2}}-V{|}_{k_{inj}^{\prime }}^{n-\frac{1}{2}}+V_{s}{|}^{n-\frac{1}{2}}) \end{array}$$

(17) $$\begin{array}{cc} V{|}_{k_{inj}^{\prime }}^{n+\frac{1}{2}} & =V{|}_{k_{inj}^{\prime }}^{n-\frac{1}{2}}-Z_{0}C_{u}(I{|}_{k_{inj}^{\prime }+\frac{1}{2}}^{n}-I{|}_{k_{inj}^{\prime }-\frac{1}{2}}^{n}+\frac{V_{s}{|}^{n}}{Z_{0}}). \end{array}$$

The transmission line is terminated at $k^{\prime }=0$ with a first-order absorbing boundary condition [40]

(18) $$V{|}_{0}^{n+\frac{1}{2}}=V{|}_{1}^{n-\frac{1}{2}}+\frac{C_{u}-1}{C_{u}+1}(V{|}_{1}^{n+\frac{1}{2}}-V{|}_{0}^{n-\frac{1}{2}}).$$

Between $k^{\prime }=0$ and $k_{exc}^{\prime }$, we define the observation point $k_{obs}^{\prime }$, where we record the voltage and current of the line.

The transmission line interacts with the FDTD grid at the antenna feed point; the electric field at the antenna feed gap is determined by ${V|}_{k_{top}^{\prime }}$ at the line termination point,

(19) $$E_{z}{|}^{n+\frac{1}{2}}=-\frac{V{|}_{k_{top}^{\prime }}^{n+\frac{1}{2}}}{\delta h}.$$

The transmission-line current ${I|}_{k_{top}^{\prime }+\frac{1}{2}}^{n}$ is determined by the line integral of *H* around the feed point, similar to the feed-gap models. The antenna voltage and current are given by $V_{a}{=V|}_{k_{top}^{\prime }}^{n+\frac{1}{2}}$ and $I_{a}{=I|}_{k_{top}^{\prime }+\frac{1}{2}}^{n}$, respectively.

The interaction in the FDTD simulation occurs at $k_{top}^{\prime }$, whereas the incident and reflected waves are recorded at positions $k_{inj}^{\prime }$ and $k_{obs}^{\prime }$. The time it takes for the waves to traverse the transmission line should be accounted for when computing the frequency response of the FDTD signals. We normalize the frequency response of all signals by the time-shifted exciting signal ${\hat{V}}_{s}e^{-j\omega \delta t_{inj}}$, with $\delta t_{inj}=\left(k_{top}^{\prime }-k_{inj}^{\prime }\right)\delta hu_{p}^{-1}$. Similarly, the observed signals are time-shifted to the line termination point as ${\hat{V}}_{obs}e^{j\omega \delta t_{obs}}$, with $\delta t_{obs}=\left(k_{top}^{\prime }-k_{obs}^{\prime }\right)\delta hu_{p}^{-1}$. For the computation of ${\hat{G}}_{b}$, we set ${\hat{I}}_{s}$ equal to the ${\hat{I}}_{a}$ of the Tx antenna.

#### 2.2.4. Comparison of Antenna Modeling Accuracy

**Figure 6:**
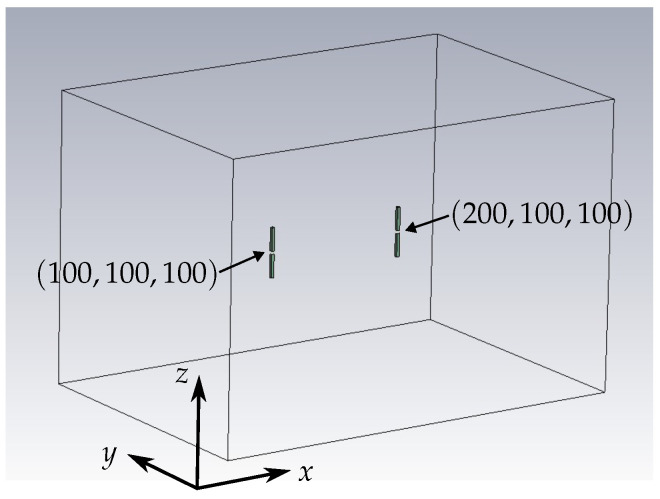
Validation setup for comparing antenna models, based on calculating transmission between two identical elements immersed in a dispersive medium.

We validated the developed antenna models by simulating simple scenarios using CST Microwave Studio. The first scenario consisted of a box of size 300 mm × 200 mm × 200 mm, filled with a 90% glycerol–water mixture, and two dipole antennas centered at points (100,100,100) and (200,100,100) oriented parallel to the *z* axis (Figure 6). We created two additional scenarios by replacing the dipoles with the planar-spear and patch-spear antennas. We modeled the dipoles as PEC structures in CST. For the planar-spear-monopole antenna, we modeled the metallic parts as pure copper and the substrate as 2.24 mm thick FR4 ($\epsilon_{r}=4.3$, tanδ=0.025). The metallic parts of the spear-patch antenna were also modeled as pure copper, while the substrate consisted of 3.5 mm thick lossless alumina ($\epsilon_{r}=9.9$). We used discrete ports terminated at 50 Ω as antenna feeds and the CST time-domain solver to simulate the models.

As a figure of merit, we compare the transmission coefficient $S_{12}$ obtained from CST Microwave Studio with our implementation. The computation of the *S*-parameters depends on the feed model used for the antenna. In particular, extracting the values from the transmission-line model is straightforward. For the *q*-th Tx and *p*-th Rx pair,

(20) $$S_{pq}=\frac{{\hat{V}}_{obs,q}e^{j\omega \delta t_{obs}}}{{\hat{V}}_{s,p}e^{-j\omega \delta t_{inj}}}.$$

For the gap feed model, we obtain the voltage and current in the antenna gap, which are converted into incident and reflected waves in a fictitious feeding line [41]. First, we compute the antenna impedance $Z_{a}={\hat{V}}_{a}{\hat{I}}_{a}^{-1}$ and the reflection coefficient for a virtual transmission line,

(21) $$\Gamma =\frac{Z_{a}-Z_{0}}{Z_{a}+Z_{0}}$$

where $Z_{0}$ is the characteristic impedance, as in the transmission-line model. For a given antenna, the incident wave relates to the antenna voltage as ${\hat{V}}_{a}{\left(1+\Gamma \right)}^{-1}$, and the reflected wave as ${\hat{V}}_{a}\Gamma {\left(1+\Gamma \right)}^{-1}$. Hence, $S_{pq}$ is computed as

(22) $$S_{pq}=\frac{\Gamma_{p}\left(1+\Gamma_{q}\right)}{1+\Gamma_{p}}\frac{{\hat{V}}_{a,p}}{{\hat{V}}_{a,q}}.$$

For all simulations, we used δh=2 mm and $\delta t=\delta h{\left(2c\right)}^{-1}$, where *c* is the speed of light. We set the length of the transmission line to 300 mm, $Z_{0}=50\;\Omega$, and $u_{p}=c$. We ran the FDTD and CST Microwave Studio simulations for the same duration, which was at least 10.5 ns.

### 2.3. Image Reconstruction

#### 2.3.1. Reconstruction Experiments

**Figure 7:**
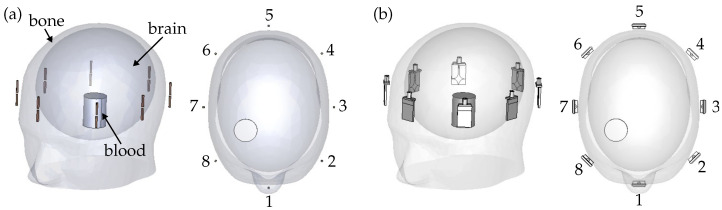
Side view (left) and bottom view (right) of the SAM head model comprising two layers, bone and brain, and a cylindrical target, modeled as blood. The SAM head model is surrounded by (**a**) an eight-dipole-antenna array and (**b**) an eight-element spear-monopole array or an eight-patch-spear-antenna array.

We assessed the impact of the level of detail in the antenna model on the reconstructions of a numerical head model depicted in Figure 7. In this simulated imaging scenario, “measured” data correspond to the *S*-parameters calculated using CST Microwave Studio. To isolate the impact of antenna modeling from other uncertainties, we consider an ideal scenario in which the structure of the head model (except the target) is known. This known head structure is used both as an initial guess and as a reference to calibrate the mismatch between the CST Microwave Studio data and the data generated by our forward FDTD solver, as described in Section 2.1.4. The numerical setup makes it possible to study the effect of antenna model complexity on image reconstruction accuracy, isolated from other model mismatch errors or noise that one may encounter in experimental setups.

We performed all reconstructions with δh=2 mm, running 10 DBIM iterations with λ=0.1 for the regularization parameter. We used $N_{\omega }=5$ frequencies, namely 0.8 GHz, 0.9 GHz, 1.0 GHz, 1.1 GHz and 1.25 GHz. The TwIST algorithm was executed for up to 125 iterations or until the relative decrease in the regularized LLS objective function fell below $e_{r}=0.006$ for the dipole and the spear monopole and below 0.09 for the patch-spear antenna. In the latter case, a scaling factor of 0.54 in the δϵ update yielded the best reconstruction performance. Forward simulations were executed until 11.67 ns, which was sufficient for the signals to traverse the domain. The remaining parameters for the forward solver were set as described previously.

If an antenna is modeled as an equivalent point source in the forward solver, the placement of the point source relative to the physical antenna strongly affects the resulting near-field distribution and, consequently, the quality of the image reconstruction. The point source, as a perfectly isotropic radiator, cannot fully reproduce the electromagnetic fields generated by a finite-size antenna with spatially distributed currents and directional radiation characteristics. The objective of the point-source approximation, therefore, is not to replicate the fields produced by the actual antenna, but rather to serve as a simplified antenna model that reproduces as closely as possible the dominant near-field characteristics within the imaging region. Hence, the equivalent point-source location must be selected to maximize near-field agreement between the original antenna and the point-source model.

For the symmetric dipole antenna, the equivalent point source is placed at the feed point. This choice is justified by the geometrical and current symmetry of the dipole: the excitation current is distributed symmetrically around the feed, which coincides with the electromagnetic center of the antenna. As a result, the near-field distribution is centered at the feed location, making it the natural reference point for the point-source approximation. For the monopole and patch antennas, however, the effective radiating center does not coincide exactly with the feed because of the asymmetric current distribution and complex antenna geometry. Therefore, the optimal point-source placement was determined numerically through near-field matching. Candidate source locations were evaluated along the antenna symmetry axis normal to the antenna aperture, starting from the feed point and extending in the main radiation direction (dash-dotted line in Figure 3b,c). A displacement of zero corresponds to the antenna feed location.

The optimal placement was defined as the position that best matches the near-field distribution of the full antenna model with that of the calibrated point source. Specifically, the matching criterion was based on the error in the *z* component of the electric field, $|E_{z,ant}-{\tilde{E}}_{z,pnt}|$, where ${\tilde{E}}_{z,pnt}$ denotes the calibrated point-source field, normalized similarly to Equation (9) using a single calibration point within the imaging domain. The minimum error was obtained for source displacements of 11 mm and 16 mm from the feed for the monopole and patch antennas, respectively, indicated by the red dot in Figure 3b,c. These optimal locations were used in all reconstructions employing the point-source approximation.

#### 2.3.2. Numerical Imaging Setup

We generated simulation data with CST Microwave Studio using an eight-antenna array surrounding a Specific Anthropomorphic Mannequin (SAM) head model [34]. We incorporated an ellipsoidal structure into the head model representing the brain, consisting of white and gray matter. We also added a cylindrical inclusion with a radius of 15 mm and a height of 40 mm in the upper-left quadrant of the brain to mimic a hemorrhagic stroke. We placed the eight-antenna array around the head at equally spaced angular positions. The imaging setups, with the antennas surrounding the SAM head model, are depicted in Figure 7.

We used the dielectric properties of cortical bone, white matter, gray matter, and blood from [42] in the 0.5 GHz to 2.0 GHz frequency range with a frequency step of 75 MHz. We immersed the setup in a 500 mm × 300 mm × 270 mm box filled with the 90% glycerol–water mixture. We first simulated dipole antennas fed with a discrete port parallel to the *z* axis, followed by spear-monopole and patch-spear antennas fed with a waveguide port.

#### 2.3.3. Reconstruction Accuracy Metric

We assess reconstruction quality using the relative reconstruction error $e_{e}$ between the reconstructed geometry and the geometry containing the target,

(23) $$e_{e}=\frac{\sqrt{\sum_{n=0}^{N_{\omega }-1}\sum_{m=0}^{M-1}{\left[\epsilon_{s}\left(r_{m},\omega_{n}\right)-\epsilon_{b}\left(r_{m},\omega_{n}\right)\right]}^{2}}}{\sqrt{\sum_{n=0}^{N_{\omega }-1}\sum_{m=0}^{M-1}\epsilon_{s}{\left(r_{m},\omega_{n}\right)}^{2}}}$$

where $\epsilon (r_{j},\omega_{i})$ is the complex relative permittivity at the spatial point $r_{j}$ at frequency $\omega_{i}$. The value of the background permittivity $\epsilon_{b}$ at the end of the DBIM iteration is an estimate of the true permittivity distribution $\epsilon_{s}$.

#### 2.3.4. Imaging Time

Using more detailed antenna models results in a more complex forward problem that requires more time to solve, increasing the overall imaging time. A considerable number of computations can be performed ahead of time, such as discretizing the antenna geometry; we do not include these tasks when measuring the increase in imaging time, since for a fixed setup, the calculation is performed once. We quantify the increase in the execution time of the forward solver due to the antenna modeling in comparison to the execution time for the point-source excitation.

We used graphics processing units (GPUs) to accelerate the imaging software [30]; therefore, we expect an increase in execution time due to additional memory transfer operations. For detailed antenna models, there is a threefold increase in the number of FDTD coefficients of the *E*-field update, and the $D_{b}$ parameter changes from a scalar quantity to a spatially varying function of *i*, *j*, and *k*. Moreover, additional computation and memory are needed to keep track of the fields $H^{I}$ and $H^{II}$ for cells containing thin PEC structures, as well as the internal states of the transmission line.

We measured all forward solver times for the SAM head model with 8 antennas, as in the image reconstruction case. We performed experiments on a desktop computer with an AMD Ryzen 5 5600X processor (AMD Inc., Santa Clara, CA, USA), 16 GB of memory, and an AMD Radeon RX 560 GPU (AMD Inc., Santa Clara, CA, USA) with 4 GB of video memory. We repeated the execution time measurements 7 times and report the median value.

## 3. Results

### 3.1. Comparison of Antenna Models

**Figure 8:**
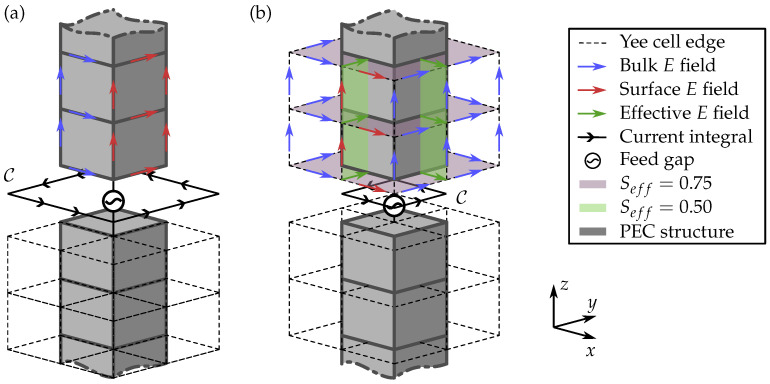
Rectangular dipole antenna models. (**a**) The dipole model is aligned with the Yee lattice. (**b**) The dipole model is centered with respect to the Yee lattice edges.

We compared two discretization approaches for dipole antennas. In the first case, the dipole body fully occupies the grid cells to align with the Yee lattice, as shown in Figure 8a. The geometry includes bulk PEC cells along the dipole, complemented by PEC surfaces on adjacent cells. We note that cells containing PEC surfaces set either the $E_{x}$ and $E_{z}$ or the $E_{y}$ and $E_{z}$ components to zero, while allowing the perpendicular *E* component to update as usual. The antenna feed gap is placed along one of the outer edges of the dipole. The aligned discretization is possible only when δh divides the dipole cross-section.

In the second, more general approach, we centered the dipole body on one edge of the Yee cell, as shown in Figure 8b. In the centered-dipole case, all cells contain both the PEC and the surrounding dielectric. Hence, we apply a conformal modeling approach and modify the permittivity and permeability accordingly. We also centered the feed gap with respect to the antenna body. In both models, we placed the current integral plane at the midpoint of the gap along the *z* direction, and the contour encompasses the entire PEC structure.

**Figure 9:**
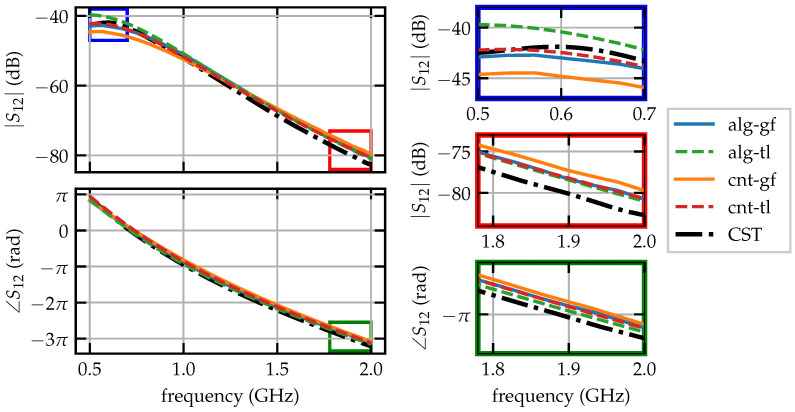
Comparison of magnitude and phase of the $S_{12}$ parameter for the FDTD dipole models vs. the CST Microwave Studio model and the setup in Figure 6. Each curve corresponds to a different FDTD antenna model: aligned-dipole model with gap feed (“alg-gf”, blue), aligned-dipole model with transmission-line feed (“alg-tl”, green), centered-dipole model with gap feed (“cnt-gf”, orange), and centered-dipole model with transmission-line feed (“cnt-tl”, red). The black curve corresponds to CST Microwave Studio simulations performed with a discrete port terminated with 50 Ω, placed as in Figure 8b. The largest $S_{12}$ differences and corresponding frequency intervals are shown in the panels on the right.

Figure 9 compares the $S_{12}$ parameter for two dipole antennas produced by our FDTD implementation under different model and feed combinations with those obtained from CST Microwave Studio simulations. The transmitted signals $S_{12}$ in Figure 9 correspond to the configuration shown in Figure 6. All geometry discretizations produce similar results, with the aligned geometry with gap feed and the centered geometry with transmission-line feed exhibiting the best match with the CST Microwave Studio results. All cases show some discrepancy with the CST-simulated $S_{12}$ at higher frequencies, most likely due to the relatively coarse grid size used in our simulations. To facilitate comparison, Table 2 presents the relative error between CST Microwave Studio and the various FDTD antenna models:

(24) $$e_{S}=\frac{\sqrt{\sum_{n=0}^{N_{\omega }-1}{\left|S_{12,CST}\left(\omega_{n}\right)-S_{12}\left(\omega_{n}\right)\right|}^{2}}}{\sqrt{\sum_{n=0}^{N_{\omega }-1}{\left|S_{12,CST}\left(\omega_{n}\right)\right|}^{2}}}$$

where $S_{12,CST}$ and $S_{12}$ are the *S*-parameters from CST Microwave Studio and from the present implementation, respectively. The values of $e_{S}$ are calculated for $N_{\omega }=45$ equally spaced frequencies between 0.5 GHz and 2.0 GHz. In the absence of discrepancies between CST and the FDTD forward solver, $e_{S}$ should be zero. Besides differences in how the antenna is modeled, discrepancies between the two models are due to meshing. The selection of a rather coarse 2 mm FDTD mesh grid may introduce slight numerical dispersion and staircase approximations of curved boundaries, accounting for the minor phase and amplitude discrepancies observed when compared to the high-fidelity conformal meshing employed by CST Microwave Studio. Table 2 also reports the relative error in magnitude $e_{\left|S\right|}$ and angle $e_{∠S}$, which were calculated similarly to Equation (24), with $S_{12,CST}$ and $S_{12}$ replaced with $20\log_{10}\left|S_{12,CST}\right|$ and $20\log_{10}\left|S_{12}\right|$, or $∠S_{12,CST}$ and $∠S_{12}$, respectively. We also calculated the relative error in the real and imaginary parts of the *S*-parameters, which we omitted from Table 2 because it was always found to be comparable to $e_{S}$.

**Figure 10:**
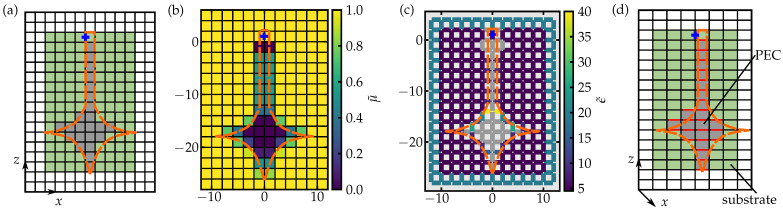
Spear-monopole antenna models. The blue cross indicates the cell edge where the feed is placed. The orange dashed line indicates the metal area on the antenna’s radiating side. All panels correspond to a side length of δh=2 mm. (**a**) Front antenna plane for model with surface constraints only. (**b**) Effective permeability for conformal antenna model. (**c**) Effective permittivity for conformal antenna model. Substrate material plotted for $\epsilon_{\infty }=4.5$ and background material plotted for $\epsilon_{\infty }=19$, with the remaining Debye parameters set to zero. (**d**) Front antenna plane for model rotated by 45° along the *z* axis. The red lines indicate the thin PEC plane’s intersection with the cell’s xy facets.

For the spear-monopole antenna, we tested three geometry discretization approaches. We composed the first case only from bulk cells and surface constraints made of either PEC or substrate material. We discretized the radiating element of the antenna as follows: we scanned the spear contour along the *z* direction in discrete δh steps and computed the PEC area. We assigned PEC material to the cell facets, while maintaining the discretized PEC area as close as possible to the area of the original shape, as shown in Figure 10a. The second approach used the conformal method to model the spear patch. Figure 10b,c plot the effective permeability and permittivity for the conformal spear model, assuming for visualization purposes non-dispersive material for the substrate ($\epsilon_{r}=4.5$) and the surrounding medium ($\epsilon_{r}=19$). The third discrete geometry is equivalent to the first model rotated by 45° along the *z* axis. Here, we used the conformal method for thin PEC structures to simulate the antenna dynamics. We discretized the radiating element contour following the same approach as in the first case, resulting in Figure 10d.

**Figure 11:**
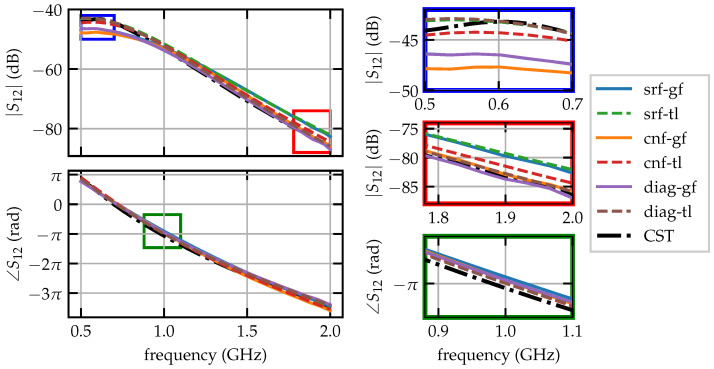
Comparison of magnitude and phase of the $S_{12}$ parameter for the spear-monopole models vs. the CST Microwave Studio model and the setup in Figure 6. Each curve corresponds to a different FDTD antenna model: surface model with gap feed (“srf-gf”, blue), surface model with transmission-line feed (“srf-tl”, green), conformal model with gap feed (“cnf-gf”, orange), conformal model with transmission-line feed (“cnt-tl”, red), diagonal surface model with gap feed (“diag-gf”, magenta), and diagonal surface model with transmission-line feed (“diag-tl”, brown). The black curve corresponds to CST Microwave Studio simulations performed with a discrete port terminated at 50 Ω, placed as in Figure 10c. The largest $S_{12}$ differences and corresponding frequency intervals are shown in the panels on the right.

Figure 11 compares the $S_{12}$ parameters for the monopole spear geometries with the CST Microwave Studio simulations. We observe good agreement among the various results, as further supported by the validation error presented in Table 2. The conformal and diagonal discretizations with the transmission-line feed provide a better match for the $S_{12}$ parameters computed by CST Microwave Studio.

**Figure 12:**
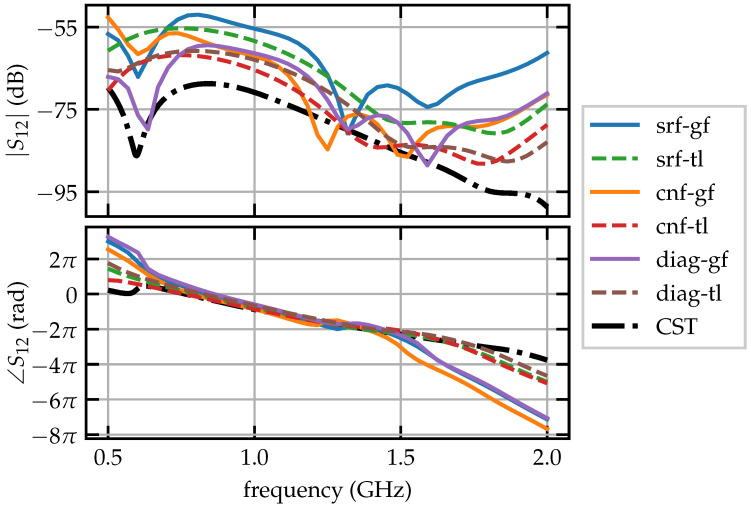
Comparison of magnitude and phase of the $S_{12}$ parameter for the FDTD patch-spear models vs. the CST Microwave Studio model and the setup in Figure 6. Each curve corresponds to a different FDTD antenna model: surface model with gap feed (“srf-gf”, blue), surface model with transmission-line feed (“srf-tl”, green), conformal model with gap feed (“cnf-gf”, orange), conformal model with transmission-line feed (“cnt-tl”, red), diagonal surface model with gap feed (“diag-gf”, magenta), and diagonal surface model with transmission-line feed (“diag-tl”, brown). The black curve corresponds to CST Microwave Studio simulations performed with a discrete port terminated at 50 Ω, placed as in Figure 10c.

Given their geometric similarity, we followed the same discretization approach for the ground-backed spear patch as for the spear monopole. The simulated transmission coefficients for the various models of the spear patch are shown in Figure 12. The discrepancy in the values of $S_{12}$ between our FDTD implementation and CST Microwave Studio was higher than for the other antenna models, as reflected in the values of $e_{S}$ in Table 2, which are an order of magnitude higher for the spear-patch antenna. This significant increase may be due to the more complex radiation properties of this antenna, which cannot be captured as accurately by our numerical modeling tools. We also note that the signal levels for the ground-backed patch antenna were over 20 dB lower than those for its monopole version due to stronger cross-polarized components, and these much lower values resulted in a more significant modeling mismatch.

### 3.2. Imaging Quality

**Figure 13:**
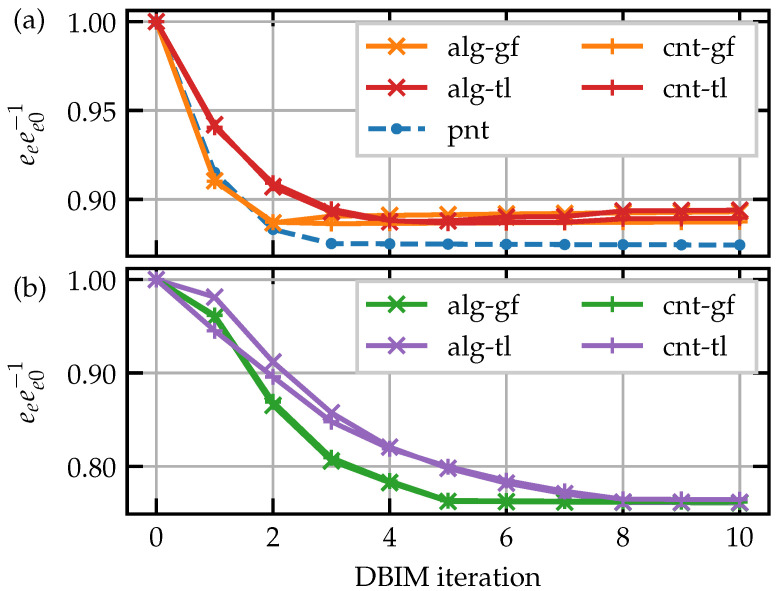
Reconstruction error versus DBIM iteration for different FDTD models of the dipole antenna in the forward solver using the CST-computed *S*-parameters as “measured data”. Each curve corresponds to a different FDTD antenna model: aligned-dipole model with gap feed (“alg-gf”), aligned-dipole model with transmission-line feed (“alg-tl”), centered-dipole model with gap feed (“cnt-gf”), centered-dipole model with transmission-line feed (“cnt-tl”), and equivalent point-source model (“pnt”). (**b**) Inverse-crime reconstruction errors for the same dipole models as in panel (**a**). CST Microwave Studio simulations were performed with discrete ports terminated at 50 Ω.

We compare the reconstruction errors for dipole antenna models of increasing complexity in Figure 13a, which plots the relative estimation error versus the DBIM iteration number. We normalized $e_{e}$ with the initial error $e_{e0}$, namely the value of $e_{e}$ for the initial guess, which is independent of the level of detail of the antenna model or the selected feed. The results in Figure 13b were produced by committing the “inverse crime” [43], where we used the forward solver to produce the “measured” values $E_{t}$ for the reconstruction. We considered the $e_{e}$ value achieved by the inverse-crime case to be the lowest attainable by the inverse algorithm for the particular imaging scenario. By establishing this “best-case” baseline, we can more effectively quantify the sensitivity of the DBIM–TwIST approach to subsequent modeling errors.

We also included the case of a point-source excitation in Figure 13a, which is the simplest choice to model a dipole with the FDTD forward solver. Interestingly, the point-source model achieved the best $e_{e}$ value among all models, despite being the least faithful representation of the dipole. For all antenna models, convergence was reached around the fourth DBIM iteration, after which the $e_{e}$ values plateaued. Models with a transmission-line feed exhibited a slower rate than the others. Inverse-crime scenarios produced lower errors, as expected, but needed more DBIM iterations to achieve a final solution.

**Figure 14:**
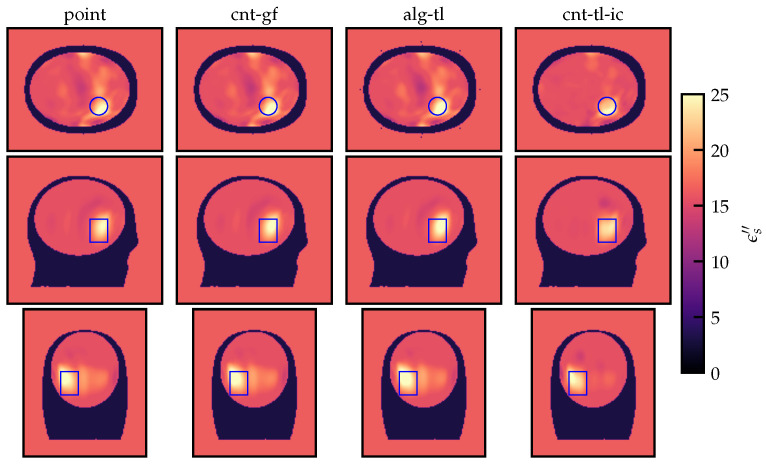
Representative slices of the reconstructed $\epsilon_{b}^{\prime \prime }$ for the setup shown in Figure 7 using dipole antennas. Rows from top to bottom show the horizontal, sagittal, and coronal planes. Columns from left to right correspond to the results with a point source (point), centered-dipole model with gap feed (cnt-gf), aligned-dipole model with transmission-line feed (alg-tl), and the inverse-crime case with centered-dipole model and transmission-line feed (cnt-tl-ic).

The small variations in errors are hardly noticeable in the reconstruction images. To illustrate this point, we present representative images of $\epsilon_{b}^{\prime \prime }$ at 1 GHz in Figure 14 for the horizontal, sagittal, and coronal planes of the reconstructed phantom. The first column corresponds to the conventional inversion with a point source, the second column to a centered-dipole model with a gap feed, the third column to the model with surface constraints excited by a transmission-line feed, and the last column to the inverse-crime case. Reconstruction with different dipole models produced visually similar results, as reflected by the comparable $e_{e}$ metric. All inverse-crime cases produced visually similar images with fewer visible artifacts than images reconstructed with the CST-produced data.

**Figure 15:**
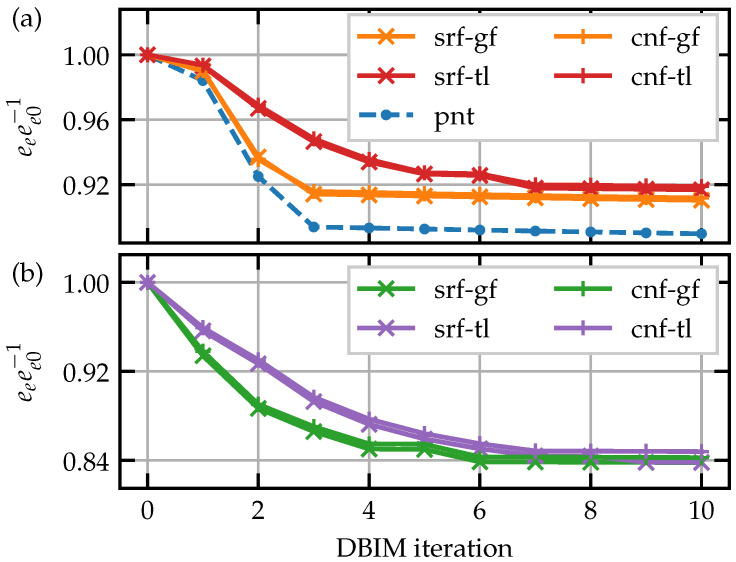
Reconstruction error versus DBIM iteration for different FDTD models of the spear-monopole antenna in the forward solver using the CST-computed *S*-parameters as “measured data”. Each curve corresponds to a different FDTD antenna model: surface model with gap feed (“srf-gf”), surface model with transmission-line feed (“srf-tl”), conformal model with gap feed (“cnf-gf”), conformal model with transmission-line feed (“cnf-tl”), and equivalent point-source model (“pnt”). (**b**) Inverse-crime reconstruction errors for the same spear-monopole models as in panel (**a**). CST Microwave Studio simulations were performed with a waveguide port terminated at 50 Ω, placed at SMA connectors on the antennas.

The $e_{e}$ results for the spear-monopole model with surface constraints and the conformal model are plotted in Figure 15. In both cases, we used the model with the conformal method for thin PEC structures for antennas 2, 4, 6 and 8. The conventional point-source model produced lower $e_{e}$ values than the spear-monopole models. Likewise, the transmission-line feed exhibited slower convergence than the gap feed across all reconstruction cases, despite being more detailed.

**Figure 16:**
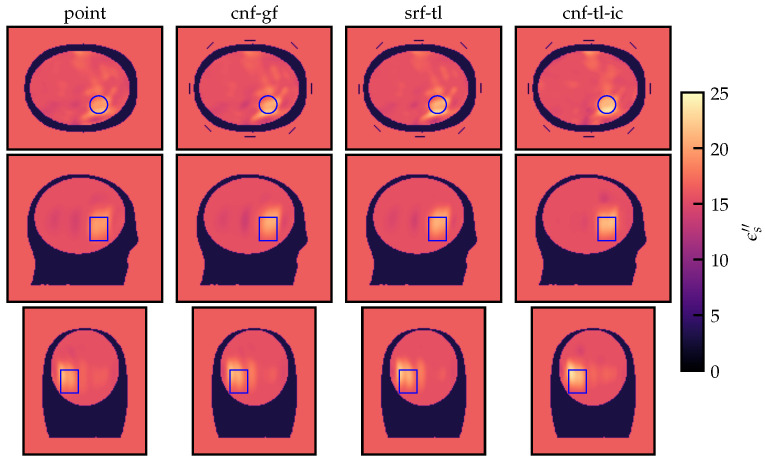
Grid with representative slices of the reconstructed $\epsilon_{b}^{\prime \prime }$ for the setup shown in Figure 7 using spear-monopole antennas. Rows from top to bottom show the horizontal, sagittal, and coronal planes. Columns from left to right correspond to the results with point source (point), spear-antenna model with conformal effective material and gap feed (cnf-gf), spear-antenna model with surface constraints and transmission-line feed (srf-tl), and the inverse-crime case with spear-antenna model with conformal effective material and transmission-line feed (cnf-tl-ic).

We present representative images produced with the spear-monopole models in Figure 16, which lead to observations similar to those obtained for the dipole antenna. Reconstructions with the point source have marginally fewer visible artifacts and better localization of the target than those performed with antenna models. Notably, the images for the spear monopoles have lower contrast compared to the dipole cases, which is evident also from the $e_{e}$ values; this observation applies both to the images resulting from the inverse-crime case and to those reconstructed using the CST Microwave Studio-simulated values.

**Figure 17:**
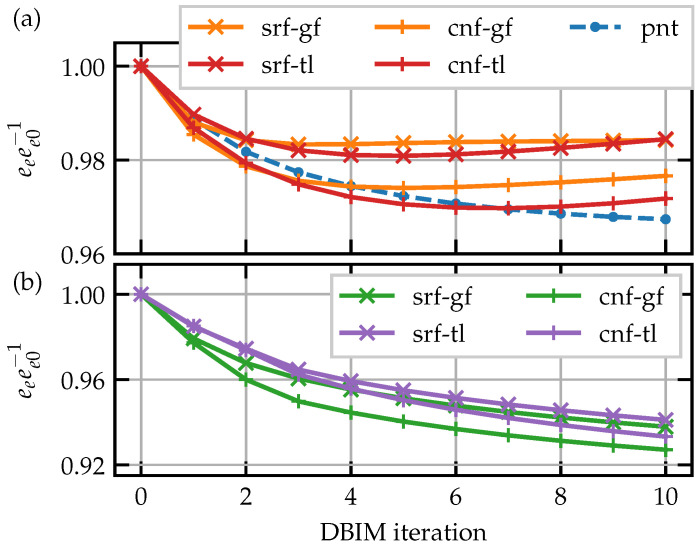
Reconstruction error versus DBIM iteration for the different FDTD models of the spear-patch antenna in the forward solver using the CST-computed *S*-parameters as “measured data”. Each curve corresponds to a different FDTD antenna model: surface model with gap feed (“srf-gf”), surface model with transmission-line feed (“srf-tl”), conformal model with gap feed (“cnf-gf”), conformal model with transmission-line feed (“cnf-tl”), and equivalent point-source model (“pnt”). Antennas at 2, 4, 6 and 8 are always modeled using the conformal method for thin PEC structures. (**b**) Inverse-crime reconstruction errors for the same spear-monopole models as in panel (**a**). CST Microwave Studio simulations were performed with a waveguide port terminated with 50 Ω, placed at the SMA connectors on the antennas.

**Figure 18:**
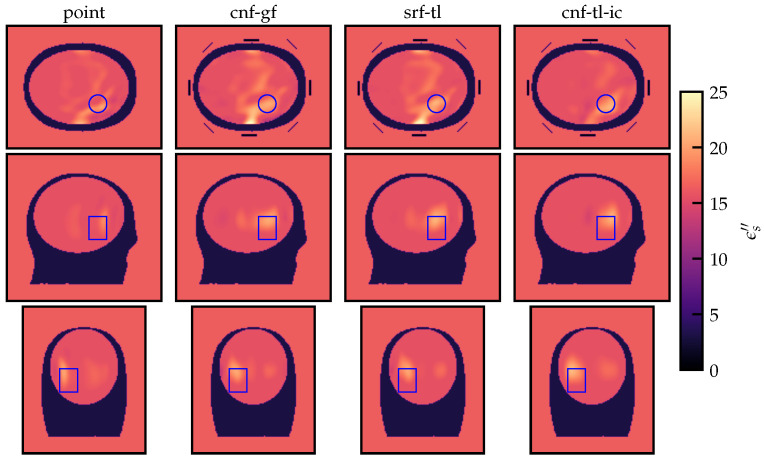
Representative slices of the reconstructed $\epsilon_{b}^{\prime \prime }$ for the setup shown in Figure 7 using ground-backed spear-patch antennas. Rows from top to bottom show the horizontal, sagittal, and coronal planes. Columns from left to right correspond to the results with a point source (point), spear-antenna model with conformal effective material and gap feed (cnf-gf), spear-antenna model with surface constraints and transmission-line feed (srf-tl), and the inverse-crime case with spear-antenna model with conformal effective material and transmission-line feed (cnf-tl-ic).

The values of $e_{e}$ for the spear-patch antennas are shown in Figure 17. Reconstruction with the conventional point-source model resulted in the lowest $e_{e}$ after 10 DBIM iterations, although the detailed antenna models exhibited faster convergence but failed to reduce $e_{e}$ after the sixth iteration. Furthermore, the antenna modeling approach affected image quality; the more detailed conformal model outperformed the surface model across all cases. Overall, the higher reconstruction errors relative to the dipole and monopole cases suggest that this non-monopole antenna is less suitable for use with the DBIM algorithm; the more severe artifacts and the lower contrast in the respective reconstructed images, as shown in Figure 18, further support this observation.

### 3.3. Modeling Error and Calibration

To quantify the impact of modeling accuracy and calibration on reconstruction accuracy, we computed the relative modeling error

(25) $$e_{t}=\frac{\sqrt{\sum_{pq\in C(8,2)}\sum_{n=0}^{N_{\omega }-1}{\left|S_{pq,t}\left(\omega_{n}\right)-S_{pq,s}\left(\omega_{n}\right)\right|}^{2}}}{\sqrt{\sum_{pq\in C(8,2)}\sum_{n=0}^{N_{\omega }-1}{\left|S_{pq,t}\left(\omega_{n}\right)\right|}^{2}}}$$

between the ground truth, $S_{pq,t}$, and the simulated, $S_{pq,s}$, *S*-parameters for the same geometry and configuration. The ground truth corresponds to the CST-computed *S*-parameters, whereas $S_{pq,s}$ results from the various FDTD antenna models. We selected the SAM model with the blood target as the scenario to assess the modeling error. The definition of $e_{t}$ is similar to Equation (24) but includes all *S*-parameters for the set of all transmitter–receiver pairs involving the eight antennas, which we denote as C(8,2). We used $N_{\omega }=45$ equally spaced frequencies between 0.5 GHz and 2.0 GHz. We note that as a rule of thumb, $e_{t}$ should be smaller than the scattered signal we wish to reconstruct.

**Figure 19:**
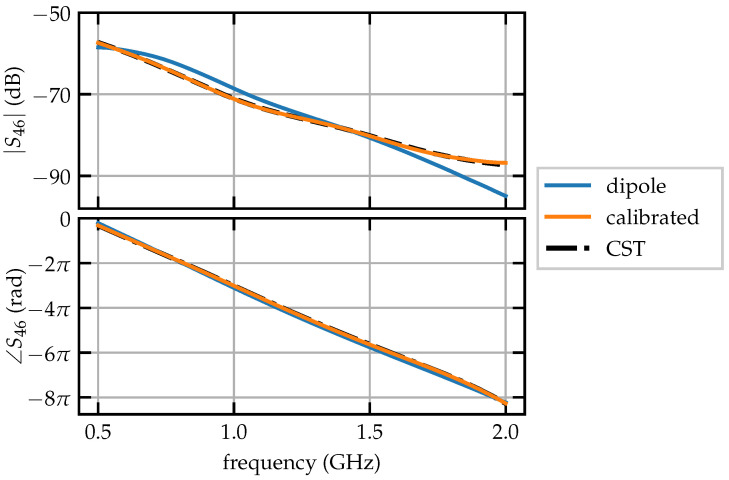
Comparison of the magnitude and phase of the $S_{46}$ parameter for uncalibrated (dipole) and calibrated (calibrated) data. The black dashed line shows the *S*-parameter calculated using CST Microwave Studio. Calibration was performed using Equation (26). Data were produced with the aligned-dipole model with gap feed.

Table 3 compares the uncalibrated values $e_{t}$ with the calibrated values $e_{t,cal}$ for the various antenna models. We computed the calibrated *S*-parameters as in Equation (9),

(26) $$S_{pq,sc}=\frac{{\hat{E}}_{t}}{{\hat{E}}_{c}}S_{pq,c}$$

and substituted $S_{pq,s}$ with $S_{pq,sc}$ in Equation (25) to calculate $e_{t,cal}$. We recall that $S_{pq,c}$ are the CST-computed *S*-parameters for the calibration geometry, and ${\hat{E}}_{t}$ and ${\hat{E}}_{c}$ are the FDTD-simulated *E* field values at the antennas for the configuration in question and the calibration geometry, respectively. The value of $e_{t,cal}$ is related to the calibrated scattered-field signal, as used in the reconstructions, for an identical scattering system and background. A comparison of the calibrated and uncalibrated *S*-parameters is shown in Figure 19 for one of the antenna pairs.

The errors for the uncalibrated signal were an order of magnitude higher than $e_{t,cal}$ and resulted from discrepancies similar to those shown in Figure 19. Moreover, the $e_{t,cal}$ values for the point-source radiator were comparable or lower than those produced by the detailed antenna models. These results agree with the observations from our reconstruction experiments and further support the claim that details in the antenna model may be redundant in the presence of calibration, which is crucial to counter the omnipresent model error that is the dominant source of reconstruction inaccuracies. The error $e_{t,cal}$ remained lower for the point source relative to the detailed models for the spear-patch antennas, despite leading to higher reconstruction errors. This suggests that the reconstruction quality degradation is due to complex wave propagation within and between the antennas that cannot be captured with a simple point-source model. We elaborate further on this point in Section 4.

In addition, we computed the relative difference in the scattered signal between the initial guess and the head model with the target using both signals from the CST Microwave Studio simulations by substituting the *S*-parameters for the initial guess instead of $S_{pq,s}$ in Equation (25). The resulting values were 0.0483, 0.0304, and 0.0498 for the dipole, spear-monopole, and patch-spear antennas, respectively. Clearly, the magnitude of the modeling error was significantly higher than the relative difference in the scattered signal, rendering the uncalibrated data impractical for imaging; numerical experiments, which we do not present here, confirmed this fact.

### 3.4. Antenna Model Impact in Cases of Reduced Total Error

Our results so far have demonstrated that more complex antenna models do not improve reconstruction quality in practical scenarios with significant model error. It is also important, however, to investigate the impact of antenna model mismatch in scenarios where we eliminate all other numerical discrepancies (such as discretization of Maxwell’s equations or dispersive material modeling) not related to antenna modeling. Moreover, it is also important to consider how the mismatch affects the results for setups in which the total reconstruction error drops, such as cases with improved spatial coverage of the imaging domain using a larger antenna array. To investigate these issues, we first studied a scenario in which the *S*-parameters produced by the FDTD forward solver using the more sophisticated conformal spear-monopole model with transmission-line feed were treated as “measured data”. To introduce an antenna model error, the reconstruction was performed using the spear-monopole model with surface constraints and gap feed, as well as the point-source excitation model. In the second scenario, we kept the measurement and reconstruction antenna model the same, but we considered two antenna rings to increase data diversity.

**Figure 20:**
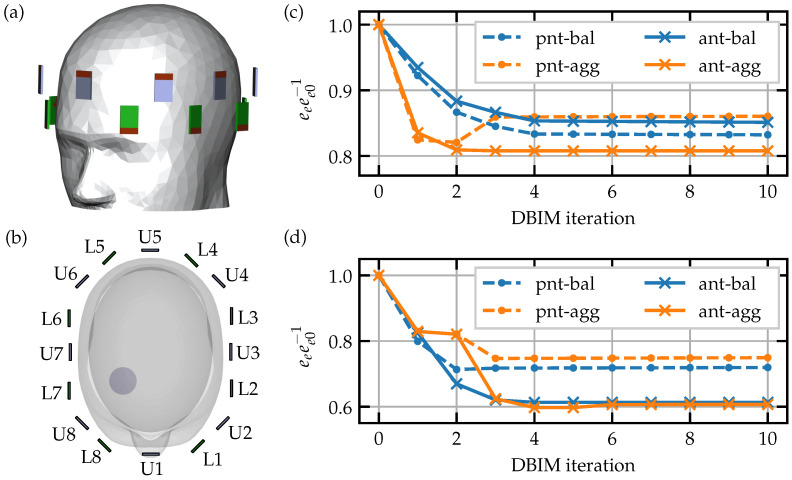
Illustration of imaging cases with reduced model error, where we use “sophisticated” spear-monopole models to produce “measured data”, and a less detailed monopole model (“ant”) or equivalent point-source model (“pnt”) for the inversion. (**a**) Side and (**b**) top views of the setup with 16 antennas arranged in two rings. The antennas are shown in purple and marked with “U” for the upper ring, and in green and marked with “L” for the lower ring. (**c**,**d**) reconstruction error versus DBIM iteration for the 8- and 16-antenna arrays, respectively. Each curve corresponds to one of the two antenna models, and “balanced” (“bal”) or “aggressive” (“agg”) settings for the DBIM–TwIST algorithm.

For these scenarios, the experiments were carried out with an 8-antenna setup, as described in Section 2.3.2, and a setup with 16 antennas arranged in two rings, as shown in Figure 20a. The placement of the upper-ring antennas in the horizontal plane is the same as that of the eight-antenna setup, but the ring has been displaced vertically by 17.7 mm. The lower ring is spaced 3 mm below; the antenna positions are offset in the horizontal plane with respect to the upper ring and fall in between the antenna positions of the upper ring, as shown in Figure 20b. All antennas are placed such that the feeds are diametrically opposite between the two rings.

Figure 20c presents the reconstruction error for the eight-antenna setup. The results marked as balanced (“pnt-bal” for the point source and “ant-bal” for the monopole model) correspond to the reconstruction with the DBIM–TwIST algorithm and the settings described in Section 2.3. As the antenna model error is low in this imaging scenario, we also tried more “aggressive” settings for the DBIM–TwIST algorithm; this search yielded λ=0.01 and $e_{r}=0.0006$ for the smallest $e_{e}$ for the monopole antenna model reconstruction. The reconstruction error for these aggressive settings is marked with “agg” in Figure 20c. For balanced settings, the images produced by the forward solver with the point source achieved a smaller value of $e_{e}$ compared to those with the monopole model, whereas the opposite applies for the aggressive DBIM–TwIST settings.

**Figure 21:**
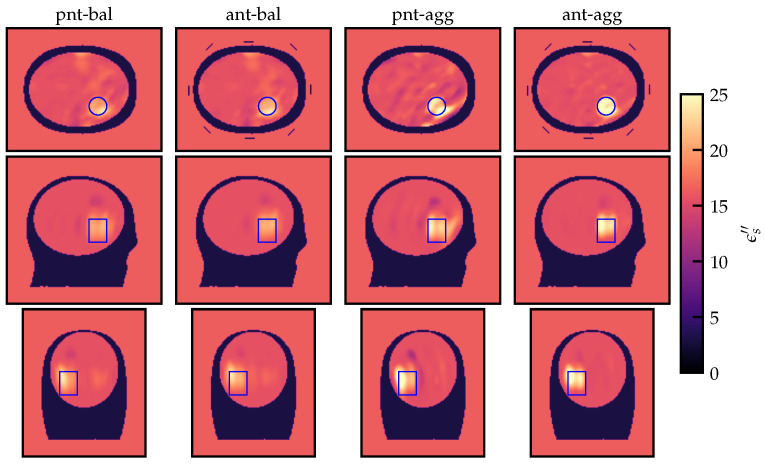
Representative slices of the reconstructed $\epsilon_{b}^{\prime \prime }$ for the cases in Figure 20 for only one ring (the same as the 8-antenna setup shown in Figure 7). Rows from top to bottom show the horizontal, sagittal, and coronal planes. Columns from left to right correspond to results with point source with balanced settings (pnt-bal), monopole model with balanced settings (ant-bal), point source with aggressive settings (pnt-agg) and monopole model with aggressive settings (ant-agg).

**Figure 22:**
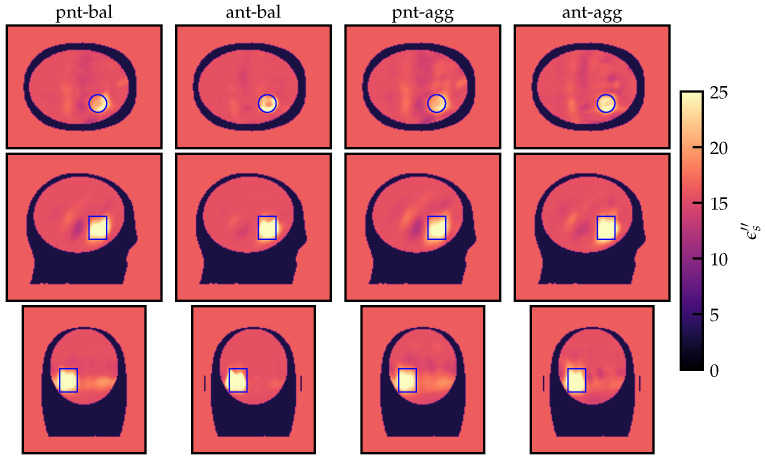
Same as Figure 21 for the 16-antenna setup shown in Figure 20.

Figure 21 and Figure 22 present corresponding images produced for these cases. For 8 antennas and balanced settings, visual differences are impossible to discern; for the aggressive settings, however, the images reconstructed with the point source exhibit more artifacts in the area surrounding the target. For the 16-antenna setup, we used two different sets of parameters to minimize $e_{e}$ for the point source and the monopole antenna model; the first set is the balanced approach with λ=0.1 and $e_{r}=0.0006$, and the latter uses the more aggressive values of λ=0.04 and $e_{r}=0.0008$. Reconstruction with the monopole model outperforms that with the point source, as the values of $e_{e}$ in Figure 20 reveal. The images produced with the monopole model better identify the boundaries of the target and contain less artifacts in the exterior area of the target compared to those produced with the point source, as is evident in the resulting images in Figure 22. Predictably, the addition of the second antenna ring significantly reduces the reconstruction error and improves the image contrast.

### 3.5. Additional Imaging Scenarios

**Figure 23:**
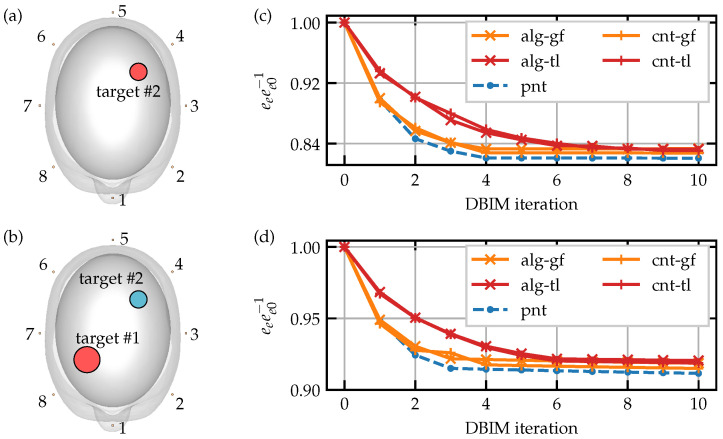
Results for the additional imaging scenarios of: (**a**) cylindrical target #2 and (**b**) two targets (labeled as target #1 and target #2). Red target color indicates blood and blue indicates CSF material. Reconstruction error versus DBIM iteration for (**c**) “target #2” and (**d**) “target #1 and target #2” and different FDTD models of the dipole antenna in the forward solver using the CST-computed *S*-parameters as “measured data”. Each curve corresponds to a different FDTD antenna model: aligned-dipole model with gap feed (“alg-gf”), aligned-dipole model with transmission-line feed (“alg-tl”), centered-dipole model with gap feed (“cnt-gf”), centered-dipole model with transmission-line feed (“cnt-tl”), and equivalent point-source model (“pnt”).

To assess whether our findings extend beyond the target considered so far to other imaging scenarios, we ran imaging experiments with the dipole antenna for two additional scenarios. In the first scenario, we used a cylinder with a radius of 10 mm made of blood, which was placed in an adjacent position compared to the original target and is marked as target #2 in Figure 23a. The second scenario included both the original target, target #1, and the smaller target, target #2, with different materials, as shown in Figure 23b. The original target was modeled as blood and target #2 as cerebrospinal fluid (CSF). The CSF material has a higher dielectric contrast than blood, with respect to the surrounding average brain material.

Figure 23c,d present the normalized $e_{e}$ for the two cases. As in the original case, the point source achieved the smallest error among all dipole models. The transmission-line feed showed a consistently slower convergence rate, with the error ending close to the gap feed models; this was also true for the “inverse crime” $e_{e}$ values. The corresponding reconstructions adequately located target #2 in all cases. For the scenario with two targets, the reconstructions did not properly identify the target #1; the smaller dielectric contrast of the blood material made it harder to discern compared to the CSF. Detection of both targets was achieved only for the inverse-crime case, independent of the antenna and feed model.

### 3.6. Imaging Times

The measured execution times for a single iteration of the forward solver and an array of eight antennas are given in Table 4, along with the relative difference compared to the point-source case. We have sorted the antenna models with increasing complexity, starting from the point-source model. The dipole model with the gap feed introduces the anisotropic FDTD coefficients and exhibits no increase in execution time. The next antenna model adds the transmission-line update, which increases the runtime by 9.61%. The spear model with surface constraints and the gap feed leads to a total increase of 23.96% compared to the point source. The conformal model with effective materials and the gap feed does not introduce additional computational complexity within the FDTD update scheme, as reflected in the execution time, which remains within the runtime of the previous configuration. The final model, which includes all the extensions discussed in Section 2.2, exhibits a relative increase in the execution time of 33.65% in comparison to the point-source model.

Overall, the complexity of the model correlates with the increase in execution time: models including only the gap-feed dipole exhibit essentially identical runtimes to the point-source model, while adding the transmission-line model and the conformal treatment of thin PEC structures increases the per-iteration runtime by roughly 10% and 24% respectively. The most sophisticated antenna representation increases the computational cost by a factor of 1.4, which can undermine its practical feasibility for large-scale imaging problems.

## 4. Discussion and Conclusions

We presented a numerical study on the impact of increasing the complexity of the antenna model in an FDTD forward solver used in MWT algorithms. To this end, we used various FDTD tools to model three different antenna types (dipole, planar monopole, and ground-backed patch antenna) and compared the transmission coefficients resulting from these models with those produced by simulating the same antennas in CST Microwave Studio. After confirming the increased modeling accuracy achieved using these techniques, we considered their impact on DBIM reconstructions of a simplified imaging scenario involving a head model and an eight-element antenna array.

Despite their added complexity, none of the proposed antenna models can represent a true experimental prototype antenna and its interaction with the interrogated domain. From this paper’s standpoint, the mismatch and its resulting model error can be classified into two categories: the first includes factors that are impossible to model, such as signals due to moving cables, fabrication tolerances such as soldering flaws, VNA measurement errors such as amplitude and phase drifts due to thermal noise, and environmental interference. Such errors can primarily be mitigated through calibration with a reference imaging scenario. The second category includes model errors that can be eliminated with very accurate numerical models; antenna model and feed fidelity are major factors in this category, which also includes accounting for material dispersion, for example.

As the model error due to the first category is dominant, MWT algorithms that include detailed antenna models also perform a calibration step [9]. Recently, advanced calibration frameworks have been proposed to de-embed antenna fidelity and other sources of model error from the object to be imaged, e.g., by treating each transmit/receive antenna pair and the intervening field propagation as a combined two-port network and calibrating scattered-field data measurements from two known targets [44]. Alternatively, the system response can be mapped by empirically defining the background Green’s function; for instance, the calibration approach in [45] demonstrated that the exact system-specific Green’s functions and illuminating incident fields can be experimentally mapped using a small, known scattering probe.

Our study suggests that the calibration step required to reduce model error is also sufficient to address the error due to approximations in the antenna model. Specifically, the point-source approximation in the FDTD forward solver was shown to be sufficient for DBIM reconstructions with CST-calculated data produced by dipole or printed monopole antennas once calibration is performed. Reconstruction with detailed antenna models exhibited some merits only for the ground-backed spear-patch antenna, which radiates in a very different way than dipoles or monopoles. Reconstructions for these antennas were less accurate due to more complex propagation and scattering effects (including antenna coupling).

Our findings, therefore, suggest that increasing the forward solver’s complexity to accurately represent the physical antenna prototypes provides limited additional benefit for monopole-type antennas, which are often deployed in MWT systems. Simple point sources are sufficient once standard calibration methods are employed. This is important considering that detailed antenna modeling increases the imaging time and software complexity, which in turn hampers code development and maintenance. Our computed reconstruction error metrics and respective imaging times in Table 4 substantiate these observations. It should also be noted that alleviating the calibration step may not be realistically possible, but calibration factors can be incorporated into the antenna model; i.e., one could fit a parametrized antenna model to the calibration data.

To consider the case of no calibration, we included inverse-crime results, which naturally led to lower reconstruction errors. However, the errors were still high for the inverse-crime scenarios because our imaging scenario of an eight-antenna ring imaging a 3D human brain is severely ill-conditioned. Hence, our observations on the small impact of antenna fidelity are also contingent upon the level of model error and ill-posedness of our imaging problem; for a better conditioned inverse problem with fewer sources of model error, antenna model inaccuracies should have a more significant effect on the reconstructions. To confirm this hypothesis, we considered additional imaging scenarios in which the “measured” data and forward solver are both produced by the same FDTD code but with a different monopole antenna model. We also added a second ring to increase data diversity and reduce the impact of regularization. The results of these cases confirmed that employing a more accurate antenna model than a point source in the forward solver can improve reconstruction quality, albeit not dramatically. In practical experimental settings, however, it would be very difficult to eliminate the model error to the level considered in these scenarios.

We also demonstrated that antenna choice affects reconstruction quality independently of how it is modeled by the forward solver. Specifically, the dipole antenna produced clearer images and a smaller reconstruction error compared to the spear-monopole antenna, both for the CST Microwave Studio-simulated measurements and in the inverse-crime case. The spear-patch antenna produced lower contrast, more pronounced artifacts, and higher localization errors compared to the other antennas. Antenna selection in MWT could be based on considering a reference imaging scenario and evaluating the resulting reconstruction error rather than relying on the $S_{11}$ parameter or other individual antenna metrics.

Clearly, our choices of antenna type and MWT implementation cannot cover the extensive range of prototypes, numerical models, or methods available in MWT. However, previous work suggests that the presented methodology and findings are applicable in a broader context. For example, the authors in [11] studied an imaging scenario similar to ours and showed that the reconstruction accuracy was similar for three different iterative MWT algorithms with two different forward solvers using the same type of calibration as in this work. This suggests that our study would lead to the same results for other electromagnetic nonlinear iterative imaging algorithms.

Moreover, results from previous work on improving the accuracy of MWT reconstructions through the use of metasurface (MTS)-enhanced antennas also agree with the present findings. For example, numerical and experimental results in [46,47] demonstrate that using MTS antennas improves reconstruction quality. The improvement was observed despite ignoring the added complexity due to the MTS in the forward model, which considered a simple point source, as in this work. The MTS improves signal coupling to the head, which translates into more accurate reconstructions even if the MTS structure is not modeled and only standard calibration is used.

To conclude, our analysis suggests that MWT experimental systems are dominated by model errors that do not depend on the sophistication with which their antennas are modeled. Improving experimental signal quality, data diversity, and calibration with respect to the MWT algorithm should be pursued regardless of the deployed antenna model. In practical scenarios, the latter can be as simple as considering a point-source excitation. This motivates a pragmatic approach to developing MWT prototypes that reduces model complexity and computational cost without sacrificing accuracy.

## Figures and Tables

**Table 1:** Antenna modeling approaches used in the present study.

	Model	“Aligned Anisotropic”	“Subcell Conformal”	“Thin PEC Surfaces”
Aspect				
Geometry boundary orientation relative to Yee cell		Aligned or staircase boundary representation	Any	Not applicable
Enforcement of PEC boundary conditions		Only for aligned PEC surfaces	Only for aligned PEC surfaces	For PEC surfaces with arbitrary orientation
Handling geometry discretization		Minimal geometry pre-processing	Complicated geometric calculations at the boundary	Identification of cells with thin surfaces
Computational complexity		Low; standard FDTD update equations	Low; standard FDTD update equations	Modified update equations; slightly higher complexity

**Table 2:** Relative error for $S_{12}$ between CST Microwave Studio and various FDTD antenna models.

FDTD Antenna Model/Feed	$e_{S}$	$e_{\left|S\right|}$	$e_{∠S}$
Dipole			
Aligned/gap feed	0.132	0.027	0.034
Centered/gap feed	0.298	0.039	0.047
Aligned/transmission-line feed	0.361	0.034	0.019
Centered/transmission-line feed	0.240	0.026	0.031
Spear monopole			
Surface/gap feed	0.331	0.043	0.045
Conformal/gap feed	0.408	0.053	0.034
Surface/transmission-line feed	0.260	0.049	0.032
Conformal/transmission-line feed	0.273	0.022	0.029
Diagonal/gap feed	0.323	0.040	0.035
Diagonal/transmission-line feed	0.167	0.013	0.030
Patch spear			
Surface/gap feed	6.423	0.379	1.430
Conformal/gap feed	5.961	0.276	1.575
Surface/transmission-line feed	4.028	0.292	0.860
Conformal/transmission-line feed	1.636	0.221	0.351
Diagonal/gap feed	2.870	0.279	1.944
Diagonal/transmission-line feed	1.958	0.224	0.771

**Table 3:** Comparison of calibrated and uncalibrated model errors.

FDTD Antenna Model/Feed	$e_{t}$	$e_{t,\mathrm{cal}}$
Dipole		
Point source	1.0010	0.0445
Aligned/gap feed	0.4428	0.0443
Aligned/transmission-line feed	0.4005	0.0442
Centered/gap feed	0.5235	0.0438
centered/transmission-line feed	0.5490	0.0438
Spear monopole		
Point source	1.0018	0.0209
Surface/gap feed	0.7979	0.0256
Surface/transmission-line feed	0.8161	0.0256
Conformal/gap feed	0.7972	0.0272
Conformal/transmission-line feed	0.8141	0.0273
Patch spear		
Point source	1.0173	0.0459
surface/gap feed	1.1945	0.0636
surface/transmission-line feed	1.1160	0.0780
conformal/gap feed	1.1845	0.0624
conformal/transmission-line feed	1.1310	0.0618

**Table 4:** Execution times for the FDTD solver.

Antenna Model/Feed	Execution Time [s]	Rel. Difference [%]
Point source	167.58	0.00
Dipole/gap feed	167.57	−0.01
Dipole/transmission-line feed	183.68	9.61
Spear-surface/gap feed	207.74	23.96
Spear-conformal/gap feed	207.73	23.96
Spear-conformal/transmission-line feed	223.97	33.65

## Data Availability

The dataset is available on request from the authors.

## Abbreviations

The following abbreviations are used in this manuscript:

3D	Three-dimensional
ADE	Auxiliary differential equation
CPML	Convolutional perfectly matched layer
CSF	Cerebrospinal fluid
DBIM	Distorted Born iterative method
FDTD	Finite-difference time-domain
GPU	Graphics processing unit
LLS	Linear least squares
MTS	Metasurface
MRI	Magnetic resonance imaging
MWI	Microwave imaging
MWT	Microwave tomography
PEC	Perfectly electric conductor
Rx	Receiver
*S*-parameters	Scattering parameters
SAM	Specific anthropomorphic mannequin
SNR	Signal-to-noise ratio
TwIST	Two-step iterative shrinkage/thresholding
Tx	Transmitter
VNA	Vector network analyzer
